# Capsaicin and TRPV1 Channels in the Cardiovascular System: The Role of Inflammation

**DOI:** 10.3390/cells11010018

**Published:** 2021-12-22

**Authors:** Sreepadaarchana Munjuluri, Dru A. Wilkerson, Gagandeep Sooch, Xingjuan Chen, Fletcher A. White, Alexander G. Obukhov

**Affiliations:** 1 The Department of Anatomy, Cell Biology & Physiology, Indiana University School of Medicine, Indianapolis, IN 46202, USA; sreepada2020@gmail.com (S.M.); druwilke@iu.edu (D.A.W.); gagansooch111@gmail.com (G.S.); 2 Institute of Medical Research, Northwestern Polytechnical University, Xi’an 710072, China; xingjuanchen@yahoo.com; 3 The Department of Anesthesia, Indiana University School of Medicine, Indianapolis, IN 46202, USA; fawhite@iu.edu; 4 Stark Neurosciences Research Institute, Indiana University School of Medicine, Indianapolis, IN 46202, USA

**Keywords:** capsaicin, TRPV1, cardiovascular disease

## Abstract

Capsaicin is a potent agonist of the Transient Receptor Potential Vanilloid type 1 (TRPV1) channel and is a common component found in the fruits of the genus Capsicum plants, which have been known to humanity and consumed in food for approximately 7000–9000 years. The fruits of Capsicum plants, such as chili pepper, have been long recognized for their high nutritional value. Additionally, capsaicin itself has been proposed to exhibit vasodilatory, antimicrobial, anti-cancer, and antinociceptive properties. However, a growing body of evidence reveals a vasoconstrictory potential of capsaicin acting via the vascular TRPV1 channel and suggests that unnecessary high consumption of capsaicin may cause severe consequences, including vasospasm and myocardial infarction in people with underlying inflammatory conditions. This review focuses on vascular TRPV1 channels that are endogenously expressed in both vascular smooth muscle and endothelial cells and emphasizes the role of inflammation in sensitizing the TRPV1 channel to capsaicin activation. Tilting the balance between the beneficial vasodilatory action of capsaicin and its unwanted vasoconstrictive effects may precipitate adverse outcomes such as vasospasm and myocardial infarction, especially in the presence of proinflammatory mediators.

## 1. Introduction

Capsaicin is a natural pungent phytotoxin, which is found in the fruits of plants from the genus Capsicum (Family: Solanaceae, Order: Solanales). Archaeological evidence from Ocampo and Tehuacan caves (now Mexico) suggests that capsaicin-containing foods, such as chili peppers, have been known to humanity since 9000–7000 B.P. [1,2]. These spicy fruits were first known only to the inhabitants of the northern part of the South American continent and then spread around the World in the 16th century after the discovery of the Americas by European explorers.

**Figure 1:**
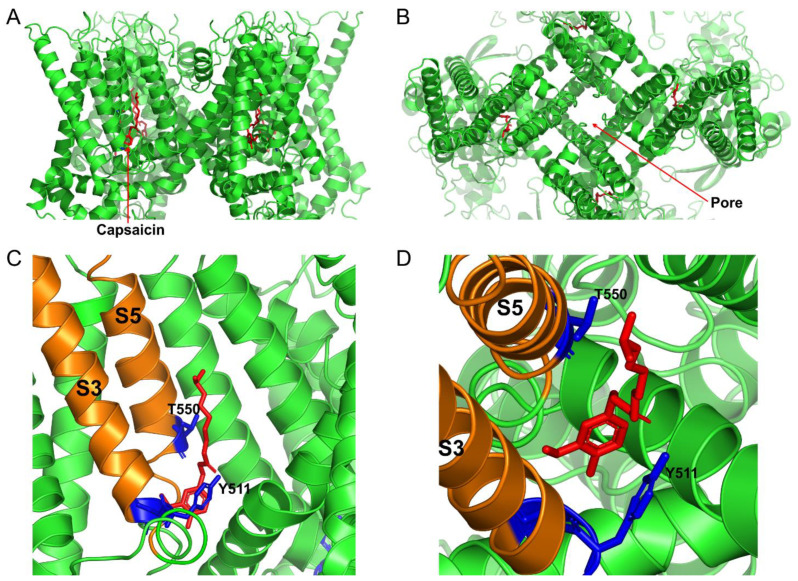
Cryo-EM structure of the open capsaicin-bound rat TRPV1 channel at 48 °C (redrawn from pdb: 7LPE, [4]). (**A**) shows the side view of the TRPV1 channel, and (**B**) shows the bird’s eye view of the channel. (**C**,**D**) show the magnified views of the capsaicin binding site from the side and from the top, respectively. The key residues that are important for coordinating capsaicin are shown in blue. S3 and S5 transmembrane domains are colored in orange, and capsaicin molecules are shown as red sticks.

Capsaicin is responsible for the “hot” burning sensation associated with consumption of the Capsicum plants and is a small hydrophobic molecule that directly activates the Transient Receptor Potential Vanilloid type 1 channel (TRPV1, formerly known as the vanilloid receptor type 1—VR1) by binding an intracellular site in the channel protein [3,4] (Figure 1). The TRPV1 protein is recognized as a molecular sensor for vanilloids, such as capsaicin, acid (pH < 6.5), and heat [5,6,7,8]. TRPV1 channels are permeable to Ca^2+^, Na^+^, and other monovalent cations. Therefore, activation of the channels results in significant Ca^2+^ and Na^+^ influx, causing cell depolarization and possibly calcium overload. Notably, prolong capsaicin-induced activation of TRPV1 leads to channel desensitization. TRPV1 exhibits a threshold to heat activation of about 43 °C [9]. Capsaicin-induced burning “hot” sensations can be alleviated by drinking cold water in humans. This is because cooling TRPV1 decreases the channel activation by capsaicin [10]. Beside heat, acid, and capsaicin, TRPV1 channels can be activated by numerous other natural hydrophobic compounds, such as 20-hydroxyeicosatetraenoic acid (20-HETE), anandamide, oxytocin, and lysophosphatidic acid (for review see [11]). Although the TRPV1 protein is predominantly found in the plasma membrane, it can also be detected in the endoplasmic and sarcoplasmic reticulum [12,13], providing a route for the release of Ca^2+^ from its intracellular stores.

**Figure 2:**
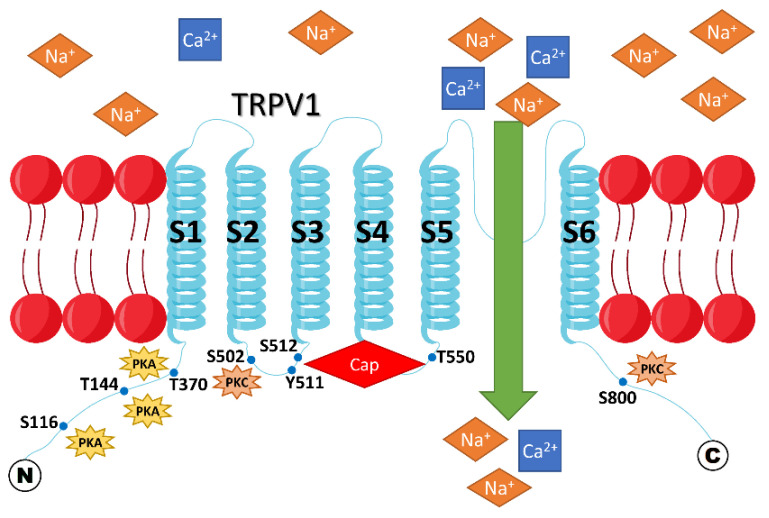
Membrane topology of rat TRPV1. The TRPV1 channel becomes sensitized if it is phosphorylated by either PKA or PKC at the indicated sites within the intracellular N- and C-termini. Sensitization of TRPV1 means that a higher cation influx through the channel is observed when it is activated by any of the natural stimuli, such as heat, H^+^, or capsaicin, compared to its unsensitized state. Cap = capsaicin.

Each TRPV1 protein is composed of four subunits that form a gated pore in the plasma membrane. Each TRPV1 subunit has six transmembrane domains and two long intracellular N- and C-termini [14] (Figure 2). The capsaicin binding site in the TRPV1 protein was mapped to the S3 and S5 transmembrane domains (Figure 1 and Figure 2), with Y511, S512, and T550 residues being identified as critical for capsaicin interaction with the rat TRPV1 protein. Capsaicin binding to the TRPV1 protein decreases the channel’s threshold temperature for heat activation [4].

**Figure 3:**
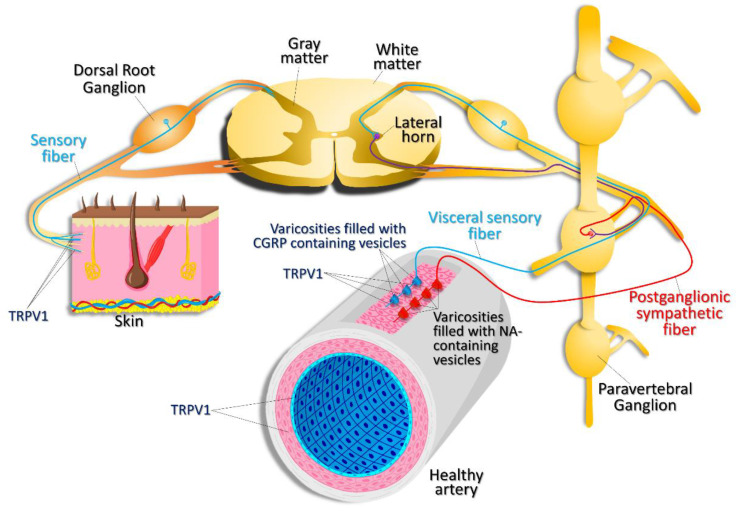
Expression pattern of TRPV1. TRPV1 channels are expressed in nociceptive, sensory nerve fibers innervating the skin and visceral sensory nerve fiber endings, smooth muscle, and endothelial cells within the vascular wall. TRPV1 activation in visceral sensory nerve endings leads to calcitonin gene related peptide (CGRP) release. CGRP then acts on vascular smooth muscle cells, causing vasodilation. Activation of postganglionic sympathetic nerve fibers results in noradrenaline (NA) release, which results in vasoconstriction.

TRPV1 channels are highly expressed in sensory nociceptive and visceral nerve fibers [15,16] (Figure 3) and are involved in inflammatory pain and hyperalgesia signaling [11]. Capsaicin and many other natural agonists of TRPV1 are known to exhibit antinociceptive properties by desensitizing TRPV1 channels on nociceptive fibers. For example, furanocoumarin imperatorin was long used in Chinese traditional medicine to relieve pain. Later, it was found that imperatorin is a partial agonist of TRPV1 [17], acting at a site adjacent to or overlapping the capsaicin-binding site of TRPV1. Imperatorin delays the recovery of TRPV1 from desensitization.

**Figure 4:**
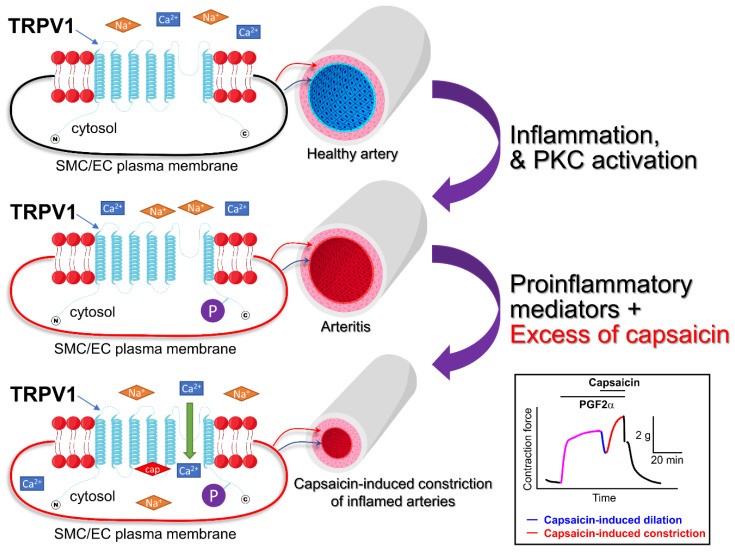
Vascular wall inflammation may sensitize TRPV1 channels expressed in vascular smooth muscle cells. PKC activity is low in healthy vessels. However, when the blood vessel becomes inflamed, the PKC is activated and PKC-dependent phosphorylation of many intracellular proteins occurs. TRPV1 channels are expressed in both endothelial and smooth muscle cells. The upper panel shows a resting smooth muscle cell (SMC)/endothelial cell (EC) in a healthy artery. In this case, the TRPV1 channel is not phosphorylated (is not sensitized). The middle panel demonstrates the phosphorylated TRPV1 channel in an inflamed vessel, capable of carrying a greater cation influx because it is sensitized. The lower panel illustrates that the excess of capsaicin may precipitate vasoconstriction overpowering capsaicin-induced dilations in the inflamed arteries. The boxed inset shows an isometric tension recording illustrating capsaicin-induced responses in a PGF2α-precontracted dog coronary artery ring [24]. Horizontal bars show the times when PGF2α and capsaicin were added to the tissue bath. cap = capsaicin.

The TRPV1 protein or RNA expression was also detected in the heart, lungs, kidneys, blood vessels, and intestine [18]. In the cardiovascular system, expression of TRPV1 has been reported in cardiomyocytes [19,20], vascular endothelial cells, and smooth muscle cells (SMCs, Figure 3 and Figure 4) [18]. However, the expression of TRPV1 in the heart is a controversial issue; there is a report indicating that TRPV1 is not expressed in cardiomyocytes [21]. On the other hand, there is strong evidence that TRPV1 is present on C- and Aδ-afferents within the epicardium [22]. It was also reported that, in endothelial cells, capsaicin can activate not only TRPV1 channels, but also the CB1 receptor [23], complicating the interpretation of in vivo data obtained by stimulating various tissues with capsaicin.

The TRPV1 channel can be sensitized via a mechanism involving the activation of G-protein coupled receptors and receptor-tyrosine kinases. Cesare and McNaughton were the first to demonstrate that heat-activated currents in nociceptive neurons can be sensitized by the bradykinin-mediated activation of a G-protein coupled receptor in a protein kinase C (PKC) dependent manner [25]. Amadesi et al. [26] further investigated the mechanisms of sensitization of TRPV1 channels under proinflammatory conditions. It was noted that inflammation is accompanied by activation of the protease-activated receptor 2, which is expressed along with TRPV1 channels, at least, in dorsal root ganglion neurons. Notably, protease-activated receptor 2-induced sensitization of TRPV1 channels involved the activation of PKC. Later, evidence was provided that TRPV1 could be tonically inhibited by phosphatidylinositol-4,5-bisphosphate (PIP_2_). PIP_2_ hydrolysis following phospholipase C activation in a G-protein coupled receptor dependent manner may relieve the channel from PIP_2_ inhibition [27,28]. These data suggested that phosphoinositide turnover may contribute to the phenomenon of sensitization of the channel. Alternatively, increased TRPV1 trafficking to the plasma membrane stimulated by nerve growth factor was also suggested as a possible mechanism for TRPV1 sensitization [29]. The mechanism was proposed to involve a direct interaction of TRPV1 and PI3K, promoting the channel translocation to the plasma membrane. In this study, evidence was provided that PIP_2_ may directly potentiate TRPV1 activity in isolated membrane patches [29]. Conversely, a dual regulation mechanism of TRPV1 by PIP_2_ and other phosphoinositides was also proposed (for review, see [30]).

Protein kinase A (cAMP-dependent Kinase, PKA), protein kinase C, and CaMKII are known to phosphorylate the TRPV1 protein at multiple sites within the N- and/or C-termini (Figure 2), reducing desensitization of TRPV1 and promoting the channel’s sensitization [31]. These residues include S116 ([31], a PKA site), T370, S502, T144, and S800 ([32,33,34], PKC sites). TRPV1 can also be phosphorylated and sensitized by tyrosine kinases [35]. Moreover, it was reported that TRPV1 should be phosphorylated by CaMKII at S502 and T704 to be sensitive to capsaicin [36]. Remarkably, inflammation is known to increase PKC and PKA activity; thus, inflammation may promote the sensitized state of the TRPV1 protein [31].

Chen et al. reported [37] that the kinetics of TRPV1 capsaicin-induced activation and desensitization in mouse dorsal root ganglion neurons can be modulated by long-term diabetic microenvironments (9 months of diabetes), augmenting the decay rate of TRPV1 responses. This study used an Ins2^+/Akita^ mouse model and proposed that, in poorly controlled type 1 diabetes, the accelerated rate of TRPV1 desensitization in dorsal root ganglion neurons may decreases TRPV1 activity and contributes to peripheral diabetic neuropathy. Thus, TRPV1 activity may be modulated by different factors influencing the channel’s desensitization.

Clinically, sensitization of the TRPV1 channel is significant because it underlies the enhanced responsiveness of nerve fibers in the inflamed tissue. Because TRPV1 is expressed predominantly in sensory fibers, which are responsible for pain and temperature sensitivity, inflammation is associated with increased pain sensations [38]. In inflamed tissue, elevated temperature may be perceived as a painful stimulus via a TRPV1 dependent mechanism, and inflammation is known to promote the phosphorylated state of TRPV1, sensitizing the channel. Notably, inflammation is a signature of many cardiovascular diseases.

Capsaicin and TRPV1 are often considered to be beneficial for cardiovascular health because capsaicin may promote weight loss, in part, by increasing the sympathetic nervous system activity, which decreases appetite and increases energy expenditure and fat oxidation [39,40]. These effects may help alleviate obesity, which is a risk factor for cardiovascular disease. There are many reviews and original research papers that only emphasize the beneficial roles of capsaicin [9,41,42,43]. Capsaicin supplements are freely available over-the-counter and many patients attempt to use them to lower weight [44,45] or to treat psoriasis [46] and other skin conditions [47]. However, a growing body of evidence suggests that, besides its beneficial potential, activation of TRPV1 by capsaicin may also lead to adverse effects [48]. In this review, we will focus on the role of TRPV1 in the cardiovascular system and discuss the beneficial and detrimental impacts that capsaicin may have on the vasculature. We will also discuss the pathophysiological role of inflammation in modulating capsaicin effects in the vasculature.

## 2. The Beneficial Roles of Capsaicin and TRPV1

Research shows that the beneficial roles of capsaicin include body weight loss, increased metabolism, painkilling, vascular dilation, etc. (for review see [49]). The benefits of capsaicin consumption or medicinal use have been studied extensively throughout the past several decades using various animal models. For example, a first possible mechanism for the anti-nociceptive effect of capsaicin was proposed by Jancsó et al. in 1977, when it was discovered that capsaicin may cause selective degeneration of a subpopulation of nociceptive neurons [50].

In 2007, Zhang et al. [51] reported that, over a period of four months, oral administration of capsaicin prevented obesity in mice in a TRPV1 dependent manner, possibly by reducing adipogenesis. This finding promoted the off-label use of capsaicin to reduce obesity in humans. However, it was noted in the report that TRPV1 expression was significantly reduced in obese male mice. This questions the effectiveness of capsaicin treatment to prevent obesity in males because of dysfunction of the capsaicin-TRPV1 dependent mechanism in the adipose tissue. In 2010, Kang et al. [52] demonstrated that dietary capsaicin-dependent activation of the TRPV1 channels and PPARα signaling in obese male mice were associated with decreased plasma levels of glucose, insulin, and leptin, as well as obesity-related glucose intolerance. These effects were in part due to reduced inflammation and increased fatty acid oxidation in the adipose tissue and liver. Additionally, Avraham et al. reported that capsaicin acting on TRPV1 channels could improve liver function in a thioacetamide-induced fulminant hepatic failure mouse model [53]. 

Later it was proposed that capsaicin may exhibit anti-cancer activity (for review see [54]). Angiogenesis is a contributing factor that promotes tumor growth. Min et al. [55] demonstrated that capsaicin decreased angiogenesis by inhibiting the vascular endothelial growth factor-induced proliferation, motility, and tube formation of primary cultured human endothelial cells by reducing the activation of p38-mitogen-activated protein kinase, FAK, and AKT. Capsaicin also suppressed angiogenesis in a chick chorioallantoic membrane assay. This occurred by preventing the continuation of the interphase process of endothelial cells, specifically cells entering the G_1_ phase. Amantini et al. [56] provided evidence that capsaicin-induced stimulation of TRPV1 can cause apoptosis of human urothelial cancer cells by activating the ataxia telangiectasia mutated/CHK2/p53 DNA damage response and Fas/CD95-mediated apoptotic pathways. The authors proposed that capsaicin treatment may help alleviate the growth of cancer cells in patients. Chen et al. also reported that capsaicin (10 mg/kg, every third day) can decrease the growth of breast cancer in a Xenograft nude mouse model in vivo by downregulating the FBI-1-NF-κB pathway [57]. However, while noting the anti-tumor potential of capsaicin, one should not disregard an earlier report by Toth and Gannett [58] demonstrating that lifelong administration of capsaicin in Swiss mice resulted in an increased incidence of benign tumors of the cecum. Notably, in this study, female mice exhibited a greater susceptibility (22%) for developing tumors compared to male mice (14%), while the control mice presented with only an 8% incidence of the same cecum tumors in both sexes. Remarkably, several earlier clinical studies also found that high intake of capsaicin might be a risk factor for gastric and esophageal cancers [59,60,61].

Neurogenic inflammation-induced cutaneous arteriole dilation was first reported in 1901 by Bayliss [62]. It was later demonstrated that neurogenic inflammation is accompanied with marked increases in cutaneous microvessel endothelial permeability and protein exudation. Both effects could be prevented by capsaicin treatment [63]. A follow-up study suggested that this effect may involve capsaicin-induced depletion of substance P from nerve terminals [64]. Because neurogenic inflammation contributes to the pathogenesis of psoriasis, Bernstein et al. [46] performed a double-blind clinical study involving 44 patients with psoriasis, which involves neurogenic inflammation. Half of the patients were instructed to apply the cream with capsaicin on their psoriasis. Many patients initially reported skin irritations upon application of the capsaicin cream, including burning, itching, and reddening of the skin. However, as patients continuously applied the cream, the irritations of the skin were reduced, including the psoriasis. This study highlighted that capsaicin may effectively treat psoriasis.

Zhong et al. [65] proposed that capsaicin may have a beneficial impact on blood pressure regulation. The authors revealed that genetic ablation of TRPV1 prevented Western diet-associated increases in nocturnal blood pressure, possibly by reducing sympathetic tone. On the contrary, TRPV1 deletion did not affect daytime blood pressure, regardless of the diet. However, later research indicated that systemic administration of capsaicin increased systemic blood pressure in mice and rats [66].

It was reported that capsaicin may play a role in preventing cardiac microvascular injury. Li et al. [67] used a type 2 diabetic mouse model induced by low-dose streptozotocin injections and high-fat diet. They found that type 2 diabetes was associated with cardiac microvascular injury. Capsaicin treatment attenuated cardiac microvascular injury, and high-glucose–high-fatty acid-induced cardiac microvascular endothelial cell apoptosis. High-glucose–high-fatty acid treatment reduced TRPV1 expression in cardiac microvascular endothelial cells and reduced their Ca^2+^ content. Cultured cardiac microvascular endothelial cells exhibited increased formation of reactive oxygen species and apoptosis if the cells were cultured in a high-glucose–high-fatty acid-containing medium; this was further aggravated by deletion of TRPV1. Thus, capsaicin may exhibit microvascular protective effects.

Finally, Ives et al. [68] reported that, in the human skeletal muscle feed arteries, TRPV1 activation opposed the α-adrenergic receptor-mediated vasoconstriction in an endothelium-dependent manner. The study concluded that TRPV1 is expressed in both the smooth muscle and the endothelial cells of human skeletal muscle feed arteries and that the channel is important for modulating the α-adrenergic receptor-mediated vasoconstriction. Interestingly, although it is not directly stated in the manuscript, these data may suggest that TRPV1 activation contributes to heat-induced flow regulation in human skeletal muscles during exercise.

Thus, there may be certain benefits of taking capsaicin-containing pills as dietary supplements. However, it should be noted that there are also drawbacks, especially for patients who present with certain inflammatory conditions, as we will discuss further.

## 3. Vascular Effects of Capsaicin

### 3.1. Vasoconstrictory Effects of Capsaicin

Studies of capsaicin effects on blood pressure and vasculature date back to the early 1980s. Donnerer and Lembeck [69] were the first to report a direct vasoconstriction induced by capsaicin in an in vivo rat model. The authors demonstrated that capsaicin exhibited a complex, triphasic blood pressure response consisting of an initial fall, followed by a slight rise and subsequent delayed fall of blood pressure without significant effects on heart rate and respiration. Their data suggested that initial and delayed falls in blood pressure were probably due to a reflex response mediated by some neuronal factors, because vagotomy decreased it. On the contrary, the observed short rise in blood pressure resulted from a direct vasoconstriction by capsaicin [69]. Pleschka et al. also found that intraarterial injections of capsaicin caused significant vasoconstriction of the tongue vessels that was apparently mediated by a direct activation of vascular receptors [70]. However, Manzini and Perretti reported that capsaicin caused vasodilation in the isolated perfused mesenteric arteries [71]. Interestingly, the authors reported that capsaicin-induced relaxation was eliminated in the presence of alpha-chymotrypsin, suggesting that such relaxation may be in part regulated by neuronally released neuropeptides such as calcitonin gene-related peptide (CGRP) [72]. CGRP acts on the CGRP receptor, which is expressed on vascular smooth muscle cells and is a complex of the calcitonin-like receptor (CLR) and a single transmembrane protein, RAMP1 [73].

The idea of a vasopressor role of capsaicin was soon contested by Pitetti et al. in 1988, who used an indirect stimulation and reported that coronary artery resistance was significantly increased when capsaicin stimulated the serosal surface of the stomach in dogs due to reflexly increased coronary α-adrenergic vasoconstriction [74], with little role for the vagus-related changes. Similarly, in 1989, Martin et al. demonstrated that capsaicin stimulation of abdominal visceral organs resulted in sympathetically mediated coronary vasoconstriction and reflexive coronary vasodilation [75]. On the other hand, in the same year, Salonen et al. provided evidence that direct application of capsaicin to the canine tracheal vasculature caused a biphasic capsaicin dose-dependent response consisting of a rapid vasoconstriction followed by a small, longer-lasting vasodilatation [76]. In 1990, Edvinsson et al. found in a cat model that capsaicin-induced cerebral vasodilatation was mediated by neuronally derived vasoactive agents and that the vasoconstrictor effect of capsaicin was most likely due to a direct effect on the cerebral vasculature [77]. In 2004, Scotland et al. proposed that myogenic vasoconstriction may occur in part due to the activation of TRPV1 on sensory C-nerve fibers innervating the mesenteric resistance arteries [78]. Scotland et al. reported that elevation of intraluminal mesenteric artery pressure led to the generation of 20-hydroxyeicosatetraenoic acid stimulating TRPV1 activity on C-nerve fiber terminals that resulted in a release of substance P, causing vasoconstriction. Thus, several initial reports attributed the vasodilatory and vasoconstrictory action of capsaicin in the vasculature to vasoactive compound release from the nerve endings (Figure 3). However, it was later found that capsaicin can directly affect blood vessels without the involvement of any neurogenic effects.

Lizanecz et al. provided evidence [79] that TRPV1 activation by capsaicin in rat skeletal muscle arterioles led to robust vasoconstriction, which was contradictory to the finding in human skeletal muscle feed arteries [68]. The authors found that capsaicin, but not anandamide, which is an endogenous TRPV1 activator, can induce significant arteriolar constriction. However, arterioles treated with anandamide exhibited no capsaicin-induced constriction due to TRPV1 cross-desensitization. On the other hand, this anandamide effect was reversed in the presence of a protein phosphatase-2B inhibitor (cyclosporin-A). Thus, PKC-dependent phosphorylation of TRPV1 modulated the channel’s sensitivity to endogenous agonists.

It was hypothesized that vascular TRPV1 channels may exhibit different properties compared to those described for TRPV1 in sensory neurons. Czikora et al. [80] reported that TRPV1 is expressed in the smooth muscles of the peripheral arteries, in which capsaicin causes vasoconstriction. The vasoconstrictive effect of capsaicin was eliminated in TRPV1 knock-out mice or in the presence of AMG9810, an antagonist of TRPV1. The authors used different agonists of TRPV1 and studied their effects on vascular TRPV1 tachyphylaxis and its vasoconstrictive potential and demonstrated that vascular TRPV1 is pharmacologically different from TRPV1 expressed in sensory nerves.

In 2014, Hiett et al. [24] were familiar with the vasodilatory effect of capsaicin in coronary arteries of pigs. The goal of their research was to delve deeper and determine the synergistic interplay between capsaicin and autocoids. They used canine conduit coronary artery rings and exposed them to varying conditions, including the following: capsaicin alone, capsaicin following pretreatment with autocoids, and capsaicin following pretreatment with autocoids to endothelium-denuded arterioles. They found that administering capsaicin alone did not have a major impact on the coronary artery rings. On the contrary, after pretreatment with autocoids, administration of capsaicin can induce some vasodilation. However, it was followed by potent capsaicin-concentration dependent vasoconstriction. Hiett et al. demonstrated that pro-inflammatory prostaglandin-thromboxane receptor agonists promoted capsaicin’s vasoconstrictory potential (Figure 4). This effect was dependent on the PLC-PKC pathway. It is likely that the PKC-dependent phosphorylation of TRPV1 induced by proinflammatory autocoids sensitized the channel, revealing its vasoconstrictive potential.

Recently, in 2020, Korishettar et al. [81] conducted a study to unveil the answer to a similar question that Hiett et al. had: does endothelin-1 regulate the vascular smooth muscle tone via TRPV1-mediated vasoconstriction? Remarkably, this group used human adipose arterioles and exposed them to increasing concentrations of capsaicin to activate TRPV1 and induce vasoconstriction. Additionally, they pre-treated some of the arterioles with endothelin-1 before challenging them with capsaicin. The authors found that the pre-treatment with endothelin-1 significantly potentiated the effects of capsaicin. These findings revealing that capsaicin-induced vasoconstrictive effects in human arterioles are particularly significant because other studies, as previously mentioned, have found links between capsaicin and vasospasm. It is known that endothelin-1 can stimulate PKC activity. The authors determined the concentration-dependence of capsaicin-induced vasoconstriction in the presence of a TRPV1 selective inhibitor, SB366791, and a PKC-selective inhibitor, GF109203X, evaluating human arteriolar constriction induced either by endothelin-1, phenylephrine, or high potassium buffer. Consistent with the report of Hiett et al., Korishettar et al. found that endothelin-1 potentiated TRPV1-elicited vasoconstriction of human adipose arterioles also in a protein kinase C-dependent manner. However, in contrast to Hiett at al., Korishettar et al. demonstrated that, in human adipose arterioles, capsaicin also stimulated the concentration-dependent vasoconstriction of untreated, resting vessels. This effect was inhibited by a TRPV1 inhibitor, SB366791, and was endothelium independent. This difference may be due to the fact that, in the human adipose arterioles, TRPV1 channels are already sensitized due to a high background secretion of pro-inflammatory adipokines by adipocytes. In both cases, endogenous compounds such as endothelin-1 and autocoids can potentiate the effects of capsaicin, possibly precipitating vasospasm. Thus, people should be taking even more caution when consuming capsaicin supplements, even though they are known to have many positive effects.

### 3.2. Vasodilatory Effects of Capsaicin

The vasodilatory effect of capsaicin was first described by Jancsó-Gábor, Szolcsányi, and Jancsó in 1970 [82] and was later confirmed by Öhlen et al. in 1987 [83] and Stjärne et al. in 1993 [84]. Jancsó-Gábor et al. used subcutaneous and intraperitoneal injections of capsaicin and observed skin blood vessel vasodilatation accompanied by hypothermia. Ohlen et al. demonstrated that topical capsaicin application resulted in potent transverse arteriole dilation. Conversely, Stjärne et al. used pentobarbital anesthetized pigs to study bilateral blood flow changes in the nasal mucosa by inserting flow-probes into both sphenopalatine arteries. Stjärne et al. compared the effects of mechanical stimulation and capsaicin on the blood flow levels in this pig model. The magnitude of the vasodilator responses was similar on both sides, although the capsaicin effect was larger as compared to the mechanical stimulation. They concluded that capsaicin infusion may evoke bilateral nasal vasodilation.

Capsaicin effects were not always associated with VR1 (TRPV1) receptor activation. Andrade et al. [23] indicated that the capsaicin vasodilatory effect may be due to the activation of the CB1 receptor, at least, in a rat aorta model. Andrade et al. found that an inhibitor of the CB1 receptor (AM281) decreased capsaicin-dependent vasorelaxation. Contrary, inhibition of TRPV1 with either capsazepine or SB-366791 or inhibition of the CB2 receptor with AM630 had no effect. Thus, analyzing in vivo effects of capsaicin, one should not overlook the possible involvement of other signaling pathways besides the TRPV1 channel activation. In addition, Gupta et al. reported [85] that capsaicin-induced relaxation of the human and porcine coronary arteries was not caused by CGRP, NK1, NO, vanilloid receptors, voltage-sensitive calcium channels, K^+^ channels, or cAMP-mediated mechanisms in an in vitro setting. However, Gazzieri et al. found that the vasodilatory effect of ethanol in concentrations that are achievable after low to moderate consumption of alcoholic beverages was attributed to activation of TRPV1 in perivascular nerve endings and consequent release of CGRP from those nerve endings [86].

TRPV1 expression on the endothelial cells has been reported by several research groups [87,88], who suggested a direct effect of capsaicin on endothelial TRPV1 channels to induce vasodilation. Yang et al. [89] reported that endothelial TRPV1 activation by dietary capsaicin promoted vasodilation and reduced blood pressure in genetically hypertensive rats. On the other hand, it was demonstrated that substance P released from nerve endings can function as a vasodilator in the model of reactive hyperemia (vasodilation after arterial occlusion). Lembeck and Donnerer [90] demonstrated that capsaicin-pretreatment caused chronic denervation reduced reactive hyperemia-associated vasodilation in the hind paw of a rat. They concluded that vasodilation might occur through the release of substance P from nerve fiber endings. Thus, although there is evidence supporting a direct effect of capsaicin on the endothelium in the vasculature, there are also other studies that demonstrate that capsaicin-mediated vasodilation occurs due to neuropeptide release from the TRPV1-expressing C-nerve fiber endings or other mechanisms. Ohlen et al. [83], who studied transverse arterioles, and Salonen et al. [76], who worked with tracheal arteries, also concluded that capsaicin-mediated dilation could be likely explained by a neurogenic mechanism involving vasodilatory neuropeptide release.

### 3.3. Capsaicin-Induced TRPV1 Activation and Atherosclerosis

Atherosclerosis is a cardiovascular disease characterized by an excessive buildup of plaques on the inner wall of large conduit blood vessels. The advanced atherosclerotic plaques consist of oxidized low-density lipoprotein (LDL), cholesterol crystals, fatty deposits, calcium phosphate, macrophages, foam cells, and proliferating smooth muscle cells. The growing atherosclerotic plaque causes the blood vessel lumen to narrow. In severe cases, atherosclerotic plaque may completely occlude a blood vessel, leading to disrupted blood flow, which may result in chronic ischemia. The rupture of the fibrous cap of an atherosclerotic plaque is an emergency if it occurs in the coronary or cerebral circulation and may precipitate myocardial infarction or stroke, which are a major cause of death in the USA. Dyslipidemia and high cholesterol are risk factors for atherosclerosis development and progression. Additionally, endothelial dysfunction and vascular smooth muscle proliferation are critical steps of atherosclerosis progression [91,92,93]. Research evidence indicates that activation of TRPV1 can regulate lipid metabolism, protects endothelial cells, inhibits smooth muscle cell proliferation, and counteract inflammation and oxidative stress [18,94,95,96].

One of the earliest studies of capsaicin’s anti-hyperlipidemic effects dates back to 1987 [97]. Negulesco et al. used a turkey model to examine the effects of capsaicin and dihydrocapsaicin on blood lipid and lipoprotein concentrations. This study compared two groups of animals. One group was maintained on a cholesterol-free diet, whereas the second group was maintained on a diet supplemented with 0.2% cholesterol. Capsaicinoids were given to treatment subgroups in both the cholesterol-enriched and the cholesterol-free groups of animals. Neither capsaicin nor dihydrocapsaicin influenced the serum triglyceride levels of the turkeys of the control diet group. However, total cholesterol (LDL-cholesterol and HDL-cholesterol) levels were increased, while VLDL cholesterol levels were decreased in the control group treated with capsaicinoids, suggesting that total cholesterol levels may be modulated and/or increased by capsaicinoids in healthy turkeys. Contrary, total cholesterol and LDL-cholesterol levels decreased with dihydrocapsaicin treatment in the group fed with a cholesterol-supplemented feed, indicating that at least dihydrocapsaicin may decrease cholesterol levels in animals exhibiting hyperlipidemia. Both compounds reduced VLDL-cholesterol and increased HDL-cholesterol in animal fed cholesterol-enriched feed. The study concluded that dihydrocapsaicin was more effective than capsaicin in mitigating hyperlipidemia in animals fed the cholesterol-enriched feed. Later, in 2006, Kiran et al. reported that capsaicin and dihydrocapsaicin affected copper-induced oxidation of human serum lipids [98]. In this study, the authors proposed that the development and progression of atherosclerosis depends on the oxidation of LDL. They decided to use human serum and test metal-induced oxidation on it rather than isolated LDL to better model the in vivo environment. After testing different concentrations, they concluded that oxidation of serum lipids is reduced by capsaicinoids in a concentration-dependent manner. However, it is unclear whether the capsaicin effects described in these studies were due to TRPV1 activation because neither selective antagonist of TRPV1 nor gene ablation was employed by the authors.

In 2009, Harper et al. [99] demonstrated that TRPV1 channels are expressed in human platelets, the aggregation of which may lead to myocardial infarction in patients with coronary atherosclerosis. The authors concluded that the release of 5-HT and ADP may be TRPV1 dependent. They provided evidence that TRPV1 may have a role in human platelets linking inflammatory mediators, such as 12- and 15-hydroxyeicosatetraenoic acid produced in atherosclerotic plaques, and platelet activation during atherosclerosis. Thus, capsaicin-induced increases in the intracellular Ca^2+^ concentration of platelets is important for their degranulation and may adversely affect patients with atherosclerosis.

Xiong et al. [100] found that dietary capsaicin may reduce endothelial dysfunction, a risk factor for atherosclerosis. The study involved ApoE^−/−^ mice. In an in vivo analysis, ApoE^−/−^ mice fed with a high-fat diet exhibited impaired coronary vasodilatation and were observed to have decreased survival rate. This phenotype was exacerbated in ApoE^−/−^ mice with genetically ablated TRPV1 and UCP2. The authors showed that capsaicin increased UCP2 expression in a TRPV1 and protein kinase A dependent manner in the endothelium and decreased mitochondrial reactive oxygen species generation. Mice fed with a high-fat diet supplemented with capsaicin exhibited a better survival. Thus, dietary capsaicin supplementation may be beneficial for preventing coronary heart disease. However, this notion has not been confirmed in any clinical studies.

### 3.4. Capsaicin and Myocardial Infarction

There are several clinical reports indicating that excessive usage of capsaicin-containing pills or painkilling patches for the purpose of weight reduction or painkilling may lead to myocardial infarction. Sogut et al. reported [101] that a healthy young male developed vasospasm and extensive inferior myocardial infarction after using cayenne pepper pills for slimming. In addition, Akçay et al. described another clinical case [102] where a 29-year-old man with no prior history of coronary artery disease developed coronary vasospasm and acute myocardial infarction induced by a prolong use of a topical capsaicin patch to treat lumbago. In this case, coronary angiography showed no atherosclerosis in either the right or the left coronary artery. Sayin et al. [103] also reported a clinical case where a 41-year-old male with no coronary artery disease used weight-loss pills containing cayenne pepper with a high content of capsaicin and developed acute myocardial infarction.

On the other hand, Imamura et al. [104] revealed that capsaicin increased the release of CGRP from cardiac C-fiber nerve endings in the heart. Notably, CGRP signaling through CLR/RAMP1 receptors was not only associated with vascular relaxation, but also might regulate proinflammatory cytokine production in dendritic cells, modulating inflammation [105]. In spontaneously beating isolated guinea pig hearts, capsaicin-induced CGRP release was associated with a marked positive inotropic and chronotropic effect. These data suggest that cardiomyocyte contractility may be increased by capsaicin. CGRP in turn stimulated histamine secretion from cardiac mast cells, and histamine is a known coronary vasoconstrictor. The release of CGRP was greater than that of histamine, weighing towards the beneficial effect of capsaicin treatment. However, the chronic use of capsaicin or large doses of capsaicin may desensitize the C-nerve fiber and eventually reduce CGRP release.

It was reported that CGRP release is endogenously elevated in acute myocardial infarction [106], peaking at about 5 h post-myocardial infarction, suggesting that CGRP may function as a local vasodilatory mediator tasked to protect the heart. Elevation of CGRP plasma concentration and capsaicin treatment was also reported to have a cardioprotective effect by Tang et al. [107]. However, again, depletion of CGRP from C-nerve fiber endings by long-term capsaicin pretreatment or a too-large capsaicin dose may decrease CGRP release and reduce the effectiveness of the endogenous protective pathway during a subsequent myocardial infarction, aggravating the outcomes. Indeed, Källner [108] demonstrated that pigs who had been treated with capsaicin had larger myocardial infarctions due to a decrease in the amount of CGRP. These results contradict the data of Tang et al. Additionally, an independent human clinical study showed that CGRP may not be elevated during acute myocardial infarction [109].

Conversely, it was demonstrated in a small cohort of 12 patients that topical administration of capsaicin via skin patches increased the ischemic threshold in patients with stable coronary disease with no change in CGRP plasma levels [110]. The authors proposed that capsaicin increased NO availability. Thus, capsaicin effects may involve different mechanisms in patients presenting with stable coronary disease or myocardial infarction.

According to a study conducted by Ren et al. [111], electrical stimulation and nociception conditioning by topical capsaicin applied to the skin in the same region in a murine model may stimulate cardioprotection and decrease myocardial infarct size. This study demonstrates that electrical stimulation through the skin will result in nociceptor-induced conditioning through the peripheral nervous system. The activation of nociceptor-induced conditioning results in the activation of bradykinin 2 receptors and activation of PKC-α. Possibly, nociceptor-induced conditioning promotes CGRP release in the heart.

Zhong et al. [112] reported that capsaicin treatment decreased infarct size, increased coronary flow, and decreased left ventricular end diastolic pressure in WT mice on a control diet. Such protective capsaicin effect was not observed in TRPV1 KO mice and was reverted by treatment with CGRP, which decreased infarct size in both strains fed with the control diet. However, the protective capsaicin effect was markedly reduced in high-fat diet-fed wild type mice. The authors concluded that the high-fat diet reduced the cardiac postischemic recovery by decreasing TRPV1 expression, increasing the likelihood of having a myocardial infarction. Because capsaicin is an activator of TRPV1 channels, this would indicate that excessive amounts of capsaicin may not be effective in those who are obese and consume a Western diet.

Conversely, Zhang et al. [113] provided evidence that chronic treatment with capsaicin causes selective degeneration of cardiac capsaicin sensitive C-nerve fibers, which are capable of releasing CGRP; this was associated with an exacerbation of the myocardial injury in acute myocardial infarction. In this study, a large dose of capsaicin was given to neonatal rats within 48 h of birth to denervate capsaicin sensitive fibers. Myocardial injury was induced at an age of about 12 weeks in male rats by ligating the left anterior descending coronary artery. Inhibition of CGRP signaling in the control group increased the infarct size. However, the authors observed that CGRP was decreased in denervated animals only during the initial stages post occlusion. At 6 h post occlusion, no difference was observed in myocardial CGRP levels between the coronary artery occlusion and the sham surgery groups in the denervated animals. This suggests that there may be additional pathways regulating CGRP release in the myocardium.

Notably, the beneficial effects of capsaicin treatment against myocardial infarction were revealed in studies using the artificial induced ischemia/reperfusion injury. It remains unclear whether capsaicin treatment would have any benefits in human patients experiencing acute myocardial infarction. The accumulated data suggest that a worsening of disease outcome is mostly reported in males, and it is unknown whether this is due to some form of sexual dimorphism.

### 3.5. Capsaicin-Induced TRPV1 Activation and Inflammation

There is contradictory information regarding the interplay of TRPV1 activation and inflammation. Inflammation is known to be associated with PKC activation. In the nervous system, the role of inflammation in regulating TRPV1 activity is well investigated. Gu et al. [114] reported that the exchange protein activated by cAMP plays a role in inflammation-induced nociception by increasing PKCα and PKCε activation and TRPV1 currents due to higher plasma membrane expression. Consistently, Joseph et al. [115] found that mice expressing a mutated TRPV1 with S801A substitution, preventing PKC-mediated phosphorylation of TRPV1 (Figure 2), exhibited impaired inflammation-mediated sensitization of TRPV1 to capsaicin, but not heat, in vivo.

The role of neuronal TRPV1 channels during inflammatory responses is complex (for review, see [116]). This may be in part because TRPV1 activation may result in the release of either pro- or anti-inflammatory signaling molecules from nerve endings. For example, Gamse et al. [117] provided evidence that capsaicin-sensitive nerve endings may release somatostatin, which has potent vasoconstrictory effects on the blood vessels in the brain [118]. Subsequently, it was reported that somatostatin released from capsaicin-sensitive nerve endings may have anti-inflammatory effects by acting via the sst4 receptor [119,120].

In the cardiovascular system, the role of TRPV1 activation in inflammation was also suggested. Sattler et al. [121] used human induced pluripotent stem cell-derived cardiomyocytes (hiPSC-CM) and found that hiPSC-CM expresses the TRPV1 channel in the plasma membrane. TRPV1 inhibition by capsazepine decreased LPS-induced IL6 release from hiPSC-CM, suggesting a role for TRPV1 activation in promoting LPS-induced cytokine release. Interestingly, LPS-induced inflammation was associated with TRPV1 internalization.

Conversely, Wang et al. [122] reported that capsaicin via TRPV1 activation increased NO production and reduced inflammation in human umbilical vein endothelial cells (HUVECs) due to increased eNOS-Ser1177 phosphorylation via a Ca^2+^- and PI_3_K/Akt-dependent signaling pathway. Capsaicin treatment also attenuated LPS-induced expression of endothelial adhesion molecules, activation of NF-κB, and monocyte adhesion in HUVECs; these effects were also dependent on TRPV1 activation. Thus, capsaicin may decrease endothelial inflammation. Consistently, Zhao et al. [123] demonstrated that activation of TRPV1 prevented oxidized LDL-induced lipid accumulation and TNF-α-induced inflammation in macrophages. In this study, the cholesterol lowering effect was due to increased cholesterol efflux via the ATP-binding cassette (ABC) transporters, ABCA1 and ABCG1, expression of which was upregulated through a liver X receptor α-(LXRα-) dependent mechanism.

Controversial data are also reported in other systems. Lee et al. [124] utilized a TRPV1 antagonist, PAC14028, as a topical cream to treat atopic dermatitis and found that it decreased expression of IL-4 and IL-13, suggesting that TRPV1 channels have proinflammatory effects. Wang et al. [125] reported conflicting evidence in mice with allergic contact dermatitis. They found that TRPV1 deficiency in mice was associated with an increase in proinflammatory markers such as TNF-α, IL-1β, and IL-6. This indicates that TRPV1 has anti-inflammatory effects. Another example of conflicting studies in terms of TRPV1 and its inflammatory versus anti-inflammatory effects include those conducted by Ninomiya et al. [126] and Wang et al. [127]. They both utilized the cecal ligation and puncture and LPS intravenous administration model to study sepsis. The first group pre-treated their septic mice with AMG9810 and capsazepine, which are both TRPV1 antagonists. They found a resultant decrease in pro-inflammatory cytokines IL-6, IL-1β, and IL-18. The second group also used TRPV1 knock-out mice, but their mice had significantly higher levels of neutrophil infiltration, serum TNF-α and IL-1β, and IL-6. There are clearly differences between how each group ran their experiments in both examples. However, their differing conclusions on TRPV1 and its relationship to inflammation suggest that there may be other TRPV1-independent mechanisms at play that are causing these contradictory results. Until these mechanisms and relationships are definitively known, it is logical for people with inflammatory conditions to avoid capsaicin as it may cause unexpected deleterious effects.

### 3.6. Pharmacokinetics of Capsaicin

Animal studies have demonstrated that capsaicin can be readily absorbed through the skin and in the gastrointestinal tract (fore review, see [128]). *Capsicum* species may contain very high levels of capsaicin. For example, Alothman et al. determined, using an ultraperformance liquid chromatography-mass spectrometry-based approach, that capsaicinoid content among *Capsicum* species is the highest in hot chilies, reaching an average amount of ~4.8 mg/g [129]. It was also shown that a single fresh fruit of *Capsicum frutescens* may contain as much as 35 mg of capsaicin [130]. Chaiyasit et al. reported [131] that the ingestion of ~27 mg of capsaicin from fresh fruits of *Capsicum frutescens* led to capsaicin blood plasma concentration of up to 8.2 nM in healthy human volunteers. They found that it takes 47 min to reach the maximum concentration of capsaicin in the blood plasma. In a cohort of 173 patients, Babbar et al. found that following topical 8% capsaicin patch administration for 1 h, the maximum systemic plasma capsaicin concentration reached up to 17.8 ng/mL (58 nM), with a mean population elimination half-life of 1.64 h [132]. Notably, Hiett et al. reported [24] that capsaicin in the nM concentration range further constricted the PGF2α-preconstricted dog coronary arteries. Thus, the high systemic bioavailability of capsaicin after either oral or topical administration points to the potential for an overdose.

## 4. Clinical Implication of Capsaicin Overuse

There is an increasing number of studies being conducted to address the safety of capsaicin use [133]. Thus, many capsaicin-containing products that were formerly highly regarded are now being looked at with some skepticism. One such example is pepper spray, a crowd control agent whose active ingredient is none-other than capsaicin. Lechner et al. [134] reported a clinical case of a 21-year-old man who was pepper sprayed and subsequently developed ventricular fibrillation. This patient had no history of underlying conditions and denied smoking, drinking, and recreational drug use. The patient was ultimately diagnosed with hypertrophic cardiomyopathy at the time of his visit, but Lechner et al. proposed that capsaicin-mediated coronary spasm leading to ventricular fibrillation might be a potential player in this case. Hence, capsaicin, despite its well-known positive effects, should be taken and used with caution because it has the potential for a disaster, as seen in this case report.

We discussed above that capsaicin treatment may result in CGRP release from cardiac C-nerve fiber endings and that this may have a cardioprotective role. However, a high dose of capsaicin causes C-nerve fiber degeneration, thus reducing its possible cardioprotective potential if capsaicin overconsumption occurs. One should note that there is a report indicating that capsaicin-induced activation of TRPV1 may stimulate perivascular afferent fibers within the myocardium to also release endothelin, which is a potent vasoconstrictor [135]. Additionally, capsaicin is likely to act on TRPV1 channels expressed on endothelial and/or vascular smooth muscle cells. Activation of TRPV1 in vascular smooth muscle cells can result in vasoconstriction, as discussed above. Phan et al. [66] demonstrated that TRPV1 is expressed in the smooth muscles of terminal arterioles in the heart, adipose tissue, and skeletal muscle of mice. In vascular smooth muscle cells, capsaicin activates a Ca^2+^ rise, which results in arteriole constriction in vivo. Indeed, Phan et al. demonstrated that systemic administration of capsaicin increased mean arterial blood pressure. In vitro, denervation of the vasculature did not reduce capsaicin-induced responses in vascular smooth muscles, thus excluding a neurogenic effect. Phan et al. found that vascular TRPV1 in arteries exhibited delayed desensitization, indicating that vascular TRPV1 may exhibit altered properties, confirming the data of other groups [24,136].

There is also growing evidence indicating that capsaicin may cause coronary spasms in patients with chronic inflammation, and that coronary spasm may lead to myocardial infarction (see also Section 3.4 in this review). A single myocardial infarction is a risk factor for consequent myocardial infarctions. Notably, severe myocardial infarction may result in death. Thus, capsaicin overconsumption may result in serious consequences. We noted already the report by Sogut et al. [101], who described a case when a healthy young male developed severe chest pain after using cayenne pepper pills that he consumed to lose weight. Inferior myocardial infarction was confirmed with electrocardiography. Illicit substances played no role, and coronary artery diseases were excluded by coronary angiogram. Thus, a likely mechanism explaining acute myocardial infarction can be vasospasm provoked by excessive capsaicin-containing pill consumption. A similar case was described by Sayin et al. [103] and Akçay et al. [137]. Consistently, coronary vasospasm and acute myocardial infarction were reported after a topical long-term capsaicin patch application [102]. Indeed, coronary artery spasm has been associated with life threatening arrhythmias and significant silent myocardial ischemia in the absence of coronary artery disease [138,139,140]. It is crucial to note that, because ethanol potentiates TRPV1-dependent heat response in the carotid artery, pepper spray, capsaicin patches, or cayenne pepper pills in combination with alcohol intoxication or cocaine syndrome may be dangerous [141]. Capsaicin-mediated mast cell activation, followed by histamine release, and histamine-induced coronary spasm may also be a factor in some cases [66].

## 5. Conclusions

Capsaicin, formulated as a topical cream or a transdermal patch, is effective for the management of pain due to minor muscle strains or cramps and joint pain due to osteoarthritis. In 2009, capsaicin was approved by the US Food and Drug Administration for treating neuropathic pain associated with postherpetic neuralgia (capsaicin 8% patch; Qutenza™). Several small clinical studies are currently underway or have been completed to determine the effectiveness of capsaicin to treat other types of pain (diabetic peripheral neuropathy, pain caused by nerve damage, nerve pain after surgery, and others) and other conditions such as rhinitis. Only a few of them have reported their results so far. For example, it has been recently demonstrated (clinical trial NCT02288156; https://www.clinicaltrials.gov, accessed on 18 November 2021) that 0.01 mM capsaicin nasal spray is effective to treat idiopathic rhinitis [142]. Additionally, modest pain relief after one 30-min capsaicin treatment (capsaicin 8% patch versus placebo patch; NCT01533428; https://www.clinicaltrials.gov, accessed on 18 November 2021) was reported in patients with painful diabetic peripheral neuropathy [143]. Brown et al. reported [144] that a single 30 min application of capsaicin 8% patch provides pain relief for as long as 12 weeks in patients with HIV-associated distal sensory polyneuropathy (NCT00321672; https://www.clinicaltrials.gov, accessed on 18 November 2021).

Several beneficial cardiovascular effects of capsaicin have also been revealed in various animal models. However, there is little or no clinical evidence supporting the notion that capsaicin may be helpful to treat cardiovascular diseases in humans. Notably, Qutenza™ is contraindicated in patients presenting with hypertension because it can increase blood pressure, either during or directly after treatment (https://www.qutenza.com, accessed on 18 November 2021).

A growing body of evidence suggests that there is a balance between vasodilatory (via the endothelium) and vasconstrictory (via the vascular smooth muscle) effects of capsaicin in the vasculature. Obesity-related inflammation promotes PKC stimulation in the vascular wall that phosphorylates TRPV1 and sensitizes the channel to capsaicin, heat, and acid action. Additionally, obesity in conjunction with elevated cholesterol facilitates arteriosclerosis progression in conduit arteries, including coronary arteries, in part by promoting clinically significant endothelial dysfunction [92,145,146,147,148,149]. Endothelial dysfunction means a reduced ability of the endothelium to release vasodilatory molecules. This may tip the balance towards the capsaicin-induced massive smooth muscle constrictions over capsaicin-stimulated endothelial-dependent dilation and may possibly precipitate vasospasm, severe arrhythmias, and/or even myocardial infarction. Thus, although we recognize capsaicin’s beneficial roles, we propose that capsaicin-containing foods/supplements should be consumed in moderation by patients with known cardiovascular disease and specifically by those who have any proinflammatory conditions. The review of the literature on this topic reveals that the majority of data on capsaicin effects have emerged from animal studies. Therefore, there is an urgent need of large-scale double-blinded clinical trials to determine whether capsaicin is beneficial for patients presenting with cardiovascular diseases.

## Data Availability

Not applicable.
